# Ensemble Models of Neutrophil Trafficking in Severe Sepsis

**DOI:** 10.1371/journal.pcbi.1002422

**Published:** 2012-03-08

**Authors:** Sang O. K. Song, Justin Hogg, Zhi-Yong Peng, Robert Parker, John A. Kellum, Gilles Clermont

**Affiliations:** 1 CRISMA Center, Department of Critical Care Medicine, University of Pittsburgh, Pittsburgh, Pennsylvania, United States of America; 2 Department of Chemical and Petroleum Engineering, University of Pittsburgh, Pittsburgh, Pennsylvania, United States of America; 3 Department of Computational and Systems Biology, University of Pittsburgh, Pittsburgh, Pennsylvania, United States of America; University of Virginia, United States of America

## Abstract

A hallmark of severe sepsis is systemic inflammation which activates leukocytes and can result in their misdirection. This leads to both impaired migration to the locus of infection and increased infiltration into healthy tissues. In order to better understand the pathophysiologic mechanisms involved, we developed a coarse-grained phenomenological model of the acute inflammatory response in CLP (cecal ligation and puncture)-induced sepsis in rats. This model incorporates distinct neutrophil kinetic responses to the inflammatory stimulus and the dynamic interactions between components of a compartmentalized inflammatory response. Ensembles of model parameter sets consistent with experimental observations were statistically generated using a Markov-Chain Monte Carlo sampling. Prediction uncertainty in the model states was quantified over the resulting ensemble parameter sets. Forward simulation of the parameter ensembles successfully captured experimental features and predicted that systemically activated circulating neutrophils display impaired migration to the tissue and neutrophil sequestration in the lung, consequently contributing to tissue damage and mortality. Principal component and multiple regression analyses of the parameter ensembles estimated from survivor and non-survivor cohorts provide insight into pathologic mechanisms dictating outcome in sepsis. Furthermore, the model was extended to incorporate hypothetical mechanisms by which immune modulation using extracorporeal blood purification results in improved outcome in septic rats. Simulations identified a sub-population (about 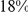 of the treated population) that benefited from blood purification. Survivors displayed enhanced neutrophil migration to tissue and reduced sequestration of lung neutrophils, contributing to improved outcome. The model ensemble presented herein provides a platform for generating and testing hypotheses *in silico*, as well as motivating further experimental studies to advance understanding of the complex biological response to severe infection, a problem of growing magnitude in humans.

## Author Summary

The pathophysiology of sepsis is complex and our mechanistic understanding remains incomplete. Mathematical models of the inflammatory response have been providing intellectual frameworks to reason about the complexity of sepsis. Due to an incompletely understood system along with very limited data, our approach focuses on building simplified, falsifiable and predictive models, and offers a means to quantify parametric uncertainty. Based on the construct that deterministic ensemble models exhibit population-like behavior, we developed a population-based computational framework that incorporates dysregulated neutrophil hyperactivity as a cellular dysfunction in septic processes. We hypothesize that probability distributions of physiological parameters conditional on population observations can characterize the range of possible physiologic responses in a population. Comparing the parameter ensembles from different phenotypes reveals some factors that play an important role in the expression of such phenotypes, such as sepsis survival. This framework can serve as an effective tool to gain insight into the pathophysiology of severe sepsis and generate testable hypotheses that guide future experiments. Our approach holds promise as a tool for integrating domain knowledge and experimental data into a quantitative assessment of population dynamics.

## Introduction

Sepsis is defined as infection accompanied by signs of systemic inflammation, such as fever, tachycardia, tachypnea, or an abnormal white blood cell count [1]. The complex pathophysiological interaction network of sepsis and its systemic nature involve many inflammatory mediators including a number of cell types, tissues and organs, making it difficult to fully understand the exact mechanisms contributing to its high mortality and morbidity despite recent progress in underlying molecular mechanisms. Furthermore, a highly variable clinical presentation significantly hampers timely diagnosis and treatment of patients with severe sepsis and adds another layer of complexity. [2], [3].

Adequate recruitment of neutrophils to sites of infection is one of the early and important features of a successful immune response. Mounting evidence suggests that severe sepsis is characterized by impaired neutrophil migration to the primary infected site and deleterious accumulation of neutrophils in distant, yet uninfected organs, resulting in organ dysfunction and death [4], [5]. Neutrophil migration to a site of bacterial infection occurs through a highly coordinated sequence of stages. Circulating quiescent neutrophils are first *primed* by interacting with inflammatory mediators that have entered the circulation as a consequence of a large local production by dedicated tissue macrophages at the site of infection and subsequent spill over of these mediators in tissue capillaries. Primed neutrophils express integrins, surface molecules that can interact with similarly activated capillary endothelial cells, resulting in rolling and *activation*. Activated neutrophils adhere to endothelial cells followed by transmigration into tissue [6], [7]. This delicate coordination is achieved through paracrine cell-cell communication, effected by chemokines and cytokines, which when present in large quantities, results in distant endocrine effects such as systemic manifestations of inflammation, distant organ endothelial activation, and overwhelming activation of neutrophils, all contributing to dysregulated neutrophil trafficking [5], [6]. Quiescent, primed, and activated neutrophils carry distinct surface molecules which can be experimentally identified, and which also play important functional roles such that specific signatures of neutrophil receptors can quantify the stage and appropriateness of the systemic inflammatory response. For example, excessive neutrophil activation leads to sequestration in distant organs and promotes tissue damage by release of reactive oxygen and nitrogen species nefarious to healthy cells [8], [9].

To better understand the pathophysiologic mechanisms involved, we developed a population-based computational framework that incorporates distinct neutrophil kinetic responses in a compartmentalized model of CLP (cecal ligation and puncture)-induced sepsis in rats. Experimentally constrained model ensembles were generated to represent a heterogeneous population, data uncertainty and other unexplained sources of variability. We previously showed that uncertain deterministic ensembles collectively exhibited population-like behavior and suggested deterministic ensembles could be a coarse-grained strategy to model population heterogeneity [10]. We explored population heterogeneity using multivariate comparative analyses of the parameter ensembles from different phenotypes (survivors and non-survivors) and identified mechanisms that may play an important role in the expression of such phenotypes.

The primary motivation of this work is the experimental observation that one form of extracorporeal blood purification, known as hemoadsorption (HA), was found to be beneficial in animal models of sepsis, including endotoxic shock [11] and CLP [12]. HA is a non-specific immunomodulatory intervention that successfully removes circulatory molecular effectors [11]. Recent evidence suggests that it may also directly impact neutrophil behavior, either by direct adsorption to the filter, or indirectly by altering immune signaling. HA is observed to decrease lung accumulation of neutrophils and improve outcome [13], but the underlying mechanisms remain elusive. The mathematical model constructed herein provides a physiologic rationale that explains such experimental observations and constitutes an *in silico* platform for generating and testing immunomodulatory interventions for sepsis. Presumably, insight as to dominant mechanisms at work would guide the rational engineering of improved HA devices resulting in an enhanced impact on outcome.

## Results

### Overview

We developed a compartmentalized, coarse-grained phenomenological model of the inflammatory response to an invading pathogen in the specific context of CLP-induced sepsis in rats. Model parameters sets were optimized to reproduce the time courses of mean plasma measurements from a cohort of septic rats, while insuring that some basic heuristic behaviors of the system in accord with published literature were maintained [14]. Because of population variability and other sources of uncertainty, we generated population-based ensemble models (*survivor* and *non-survivor* populations) which describe distinct distributions of parameter sets consistent with their experimental observations and heuristics. These ensembles were statistically generated using Markov-Chain Monte Carlo (MCMC) sampling of their posterior parameter distributions. Convergence diagnostics was applied to support that the sampling process had reached equilibrium. Prediction uncertainties in the model states were quantified over the resulting ensemble.

Simulation of the model ensembles successfully reproduced experimental observations and desired heuristic behaviors, and suggests that systemic activation of circulatory neutrophils impair their migration to primarily infected tissue, while promoting sequestration in lung tissue favoring local damage and presumably, mortality. Statistical analysis of the model ensembles obtained for separate populations provided useful insights as to key pathologic mechanisms associated with mortality in sepsis.

We next simulated a hypothetical blood purification intervention on the calibrated model ensemble. Simulations suggest that this therapy might improve targeting of primed neutrophils to the primary site of infection while interfering with lung sequestration of activated neutrophils, but that there is also a potential for harm in animal with poorly responsive immune systems.

### The dynamics of sepsis following CLP in rats

**Figure 1:**
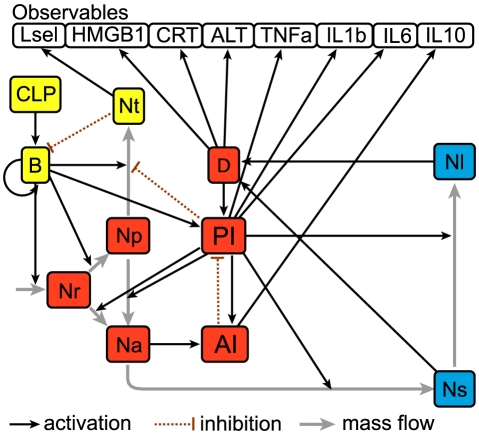
Interaction network of inflammatory responses in CLP-induced sepsis. Network nodes in different colors represent key network components in separate compartments (yellow: peritoneum, red: blood, and blue: lung). Edges represent network interactions compiled from literature (see text for details). Total 19 state variables including 8 observables. CLP, cecal ligation and puncture; B, bacteria; Nt, peritoneal neutrophil; Nr, resting blood neutrophil; Np, primed blood neutrophil; Na, activated blood neutrophil; PI, systemic pro-inflammatory response; AI, systemic anti-inflammatory response; Ns, neutrophil sequestered in lung capillaries; Nl, lung neutrophil; Lsel, L-selectin; HMGB1, High-mobility group protein B-1; CRT, creatinine; ALT, alanine aminotransferase; TNFa, tumor necrosis factor-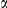; IL1b, interleukin-1 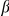; IL6, interleukin-6; IL10, interleukin-10.

We hypothesized that dysregulated neutrophil trafficking in severe sepsis may contribute to mortality [15]. We therefore developed a model of the acute innate response to an infectious challenge, with special emphasis on neutrophil trafficking and phenotypic variation. The network components and interactions were assembled based on qualitative domain knowledge of the acute inflammatory response, including multiple phenotypes of neutrophils and major effectors in three compartments: blood, peritoneum, and lung (Figure 1). To capture impaired recruitment of neutrophils, one of the key pathophysiologic features in severe sepsis, coarse-grained mechanisms influencing neutrophil migration were included in the model. In the blood compartment, neutrophils can be characterized as belonging to one of three phenotypes: resting, primed, and systemically activated. While primed blood neutrophils migrate to the site of infection and become activated locally in tissue, blood neutrophils activated in the circulation have an impaired ability to migrate to infected tissue because they possess fewer essential chemokine receptors. We chose the lung as a preferred site for the accumulation of activated blood neutrophils due to the long and narrow microvascular bed and in accord with experimental data [16]. As a result, systemically activated blood neutrophils are easily sequestered in lung capillaries. Sequestered activated neutrophils can then migrate into the lung tissue when lung vascular endothelium becomes activated by systemically circulating inflammatory mediators. The network of interactions included in the model includes 19 variables and 57 parameters. Although some parameter values were available from literature, most of them represent lumpedprocesses and therefore not directly available from published experimental studies.

Experimental data were collected from CLP-induced sepsis in rats (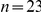), consisting of eight longitudinal measurements of key cytokines and damage-related markers in blood (see Materials and Methods). Seven rats (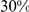) survived to seven days (the survivor population), while the remaining animals died between two and five days after CLP (the non-survivor population). In addition to the experimental data, we used the two qualitative constraints to define *survival* for *in silico* septic rats: at the end of the simulation time (200 hours), both the following constraints should be satisfied: (1) the number of bacteria (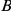) is less than 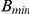 which was set to 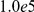 (CFU/ml), (2) the value of systemic inflammation (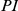) is less than 0.5 (see Materials and Methods). Otherwise rats were considered *non-survival*. To explore the ability of the model to synthesize the inflammatory responses of both the survivor and the non-survivor populations, we estimated a model ensemble separately for each population without changing model structure or initial conditions. In other words, it is assumed that the experimental settings are identical for both populations and the differences in the inflammatory responses of the two populations can be adequately represented by model parameterization.

**Figure 2:**
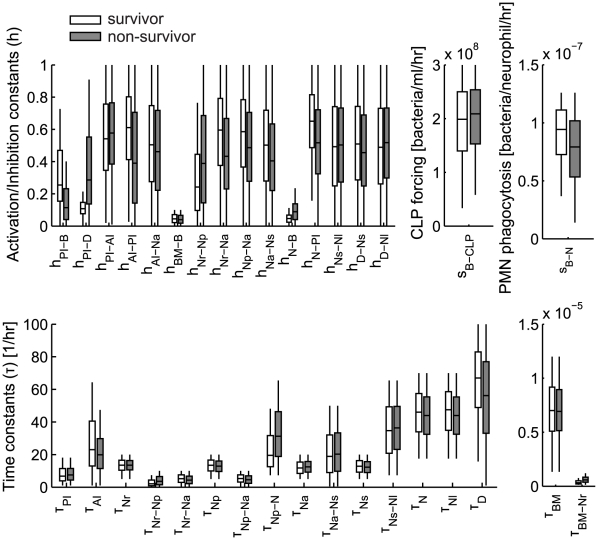
Box plot of parameter distributions for the survivor and non-survivor models.

Ensemble methods have been developed to approach ill-posed inverse problems in fields as diverse as systems biology, weather forecasting, and nuclear reaction modeling [17]–[20]. Furthermore, experimentally constrained model ensembles could capture qualitatively important network features without exact parameter information [10]. The multiple starting points for the construction of the model ensemble were constructed as initial parameter sets that produced simulations reasonably close to experimental data, while exhibiting behaviors compatible with heuristic domain knowledge (summarized in Table 1, 2). To ensure consistent observation mappings for both the survivor and non-survivor populations, only the 34 model parameters in Table 1 among the total parameters were allowed to be sampled by MCMC chains and the other parameters were kept in their baseline distribution. Five million parameter sets for each population (survivor and non-survivors) were sampled from five MCMC chains initiated from different starting points randomly chosen from the baseline parameter distribution (Figure 2).

**Table 1:** Model parameters used for the baseline case.

ID	Name	Value ( 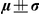 )	Unit	Description
1	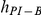	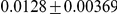	none	PI activation by bacteria
2	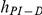	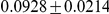	none	PI activation by damage
3	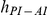	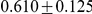	none	PI inhibition by AI
4	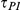	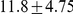	hr	PI decay
5	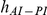	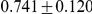	none	AI activation by PI
6	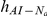	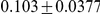	none	AI activation by 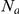
7	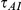	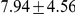	hr	AI decay
8	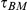	3.22e-06 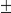 1.17e-06	hr	basal neutrophil release from bone marrow reserve
9	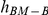	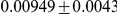	none	neutrophil release activation by bacteria
10	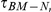	3.15e-07 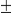 1.29e-07	hr	maximum neutrophil release from bone marrow reserve in infection
11	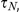	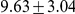	hr	resting blood neutrophil death
12	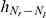	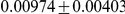	cells/ml	blood neutrophil priming by bacteria
13	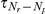	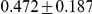	hr	blood neutrophil priming
14	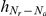	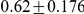	none	blood neutrophil activation by PI
15	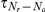	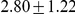	hr	blood neutrophil activation
16	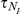	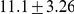	hr	primed neutrophil death
17	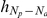	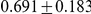	none	primed neutrophil activation by PI
18	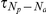	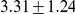	hr	primed neutrophil activation
19	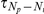	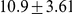	hr	neutrophil migration to tissue
20	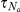	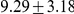	hr	activated blood neutrophil death
21	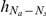	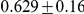	none	activation of neutrophil sequestration by PI
22	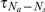	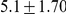	hr	neutrophil sequestration
23	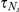	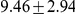	hr	sequestered neutrophil death
24	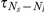	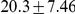	hr	primed neutrophil migration
25	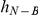	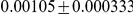	cells/ml	activation of neutrophil migration to tissue
26	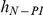	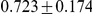	hr	inhibition of neutrophil migration to tissue
27	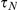	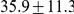	hr	tissue neutrophil death
28	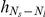	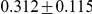	none	sequestered neutrophil migration to lung
29	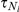	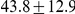	hr	lung neutrophil death
30	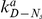	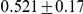	none	damage activation by sequestered neutrophil
31	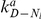	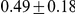	none	damage activation by lung neutrophil
32	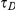	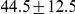	hr	damage resolution
33	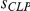	9.40e+07 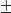 3.04e+07	numbers/ml/hr	Bacteria producing rate by CLP
34	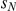	6.61e-08 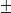 1.38e-08	ml/neu/hr	Rate of bacteria removal by migrated neutrophils

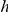
: activation/inhibition constant.

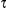
: time constant.

**Table 2:** Observation mapping parameters and other constants in the model.

ID	Name	Value ( 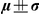 )	Unit	Description
35	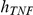	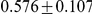	none	activating TNF 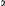 by PI
36	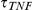	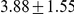	hr	TNF 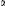 decay
37	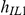	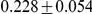	none	activating IL-1 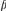 by PI
38	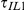	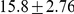	hr	IL-1 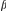 decay
39	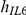	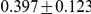	none	activating IL-6 by PI
40	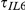	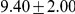	hr	IL-6 decay
41	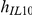	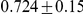	none	activating IL-10 by PI
42	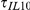	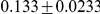	hr	IL-10 decay
43	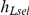	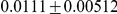	none	activating Lsel by PI
44	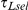	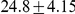	hr	Lsel decay
45	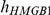	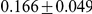	none	activating HMGB1 by PI
46	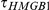	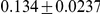	hr	HMGB1 decay
47	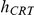	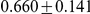	none	activating CRT by PI
48	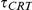	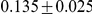	hr	CRT decay
49	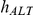	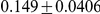	none	activating ALT by PI
50	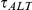	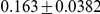	hr	ALT decay
**Constant parameters**				
51	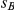	0.1	1/hr	Rate of bacterial growth
52	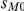	0.1	1/hr	Rate of bacterial removal by resident macrophages
53	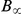	1.0e9	CFU/ml	Maximum bacteria in tissue
54	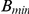	1.0e5	CFU/ml	Bacteria concentration in tissue controlled by local Macrophages (see text)
55	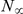	2.0e7	cells/ml	Maximum neutrophil in tissue
56	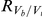	34.4/25	none	Volume ratio of blood and tissue
57	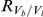	34.4/48	none	Volume ratio of blood and lung

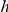
: activation/inhibition constant 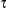: time constant.

To estimate whether stationarity had been achieved in the MCMC chains, we preformed Gelman-Rubin diagnostics, computing the potential scale reduction factor (PSRF) for each parameter [21]. The Gelman-Rubin diagnostics tests whether parallel chains converge to the same posterior distribution. PSRF is defined as the square root of the ratio of the between-chain variance and the within-chain variance. A large PSRF indicates that the between chain variance is substantially greater than the within-chain variance, so that more samples are needed. Approximate convergence was diagnosed as the PSRFs of all parameters were close to one.

**Figure 3:**
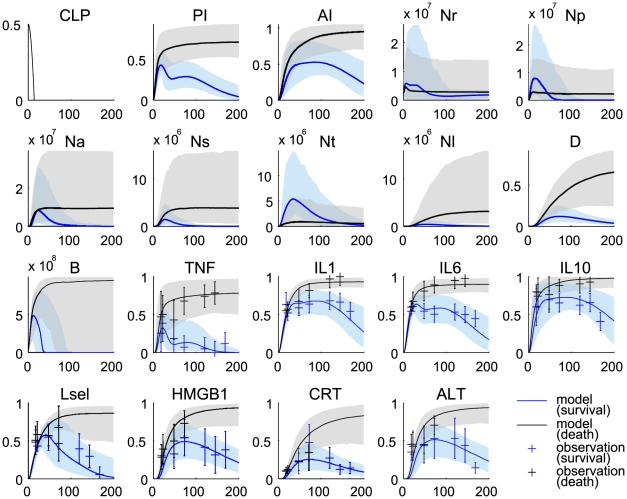
Fits and predictions of the model states. Blue denotes the survivor group and black denotes the non-survivor group. The solid lines represent the median values of the output profiles from the simulations with 1000 parameter realizations that were randomly sampled from each group of the MCMC chains (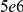 samples in each group). The shaded areas correspond to the 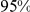 posterior limits of the model uncertainty. The error bars represent experimental observations from our septic rat experiments (normalized means and standard deviations).

The proposed model reproduced quantitative dynamic features observed in CLP-induced septic rats, verifying the description of the model reflects the inflammatory responses in CLP-induced septic rats (Figure 3). Instead of exploring explicit interactions among observation variables due to the lack of kinetic and causal information, each experimental observation was nonlinearly mapped from its high level coarse-grained state variable (see Materials and Methods). These mapping functions, which include 16 parameters (Table 2), should be consistent in both survivor and non-survivor populations and estimated in a way that minimizes the sum of cost functions for both populations. Some observation points are out of the 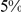
–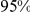 quantiles, e.g. the early time points of Lsel and ALT in 3, suggesting our proposed structure may be too simple to capture finer dynamic details of the biological process. In particular, HMGB1, CRT, and ALT are collectively used to constrain the damage state in the model. This coarse-grained approach does not allow the independent dynamic mapping of three observables from the single damage state. However, the general trends were well captured by the model, suggesting that our phenomenological description could be used to explore the general inflammatory response to CLP.

Impaired neutrophil migration to infected tissue and deleterious neutrophil accumulation in lung were predicted in the simulation of the non-survivor population. It should be emphasized that the model prediction of dysregulated neutrophil trafficking is an emergent property of the model since no neutrophil data were used to constrain model behavior (Figure 3: panels Nl, Ns, and Nt). In other words, the proposed mechanism of action about functionally heterogeneous neutrophil populations was consistent with experimental evidence not used in model training.

### Multivariate analysis of the model parameter ensembles

#### Principal component analysis

**Figure 4:**
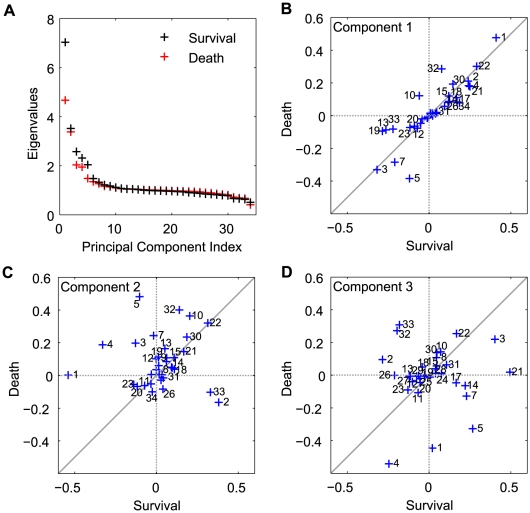
Principal component analysis of the inverse correlation matrix of the parameter ensembles for survivor and non-survivor populations. (**A**) Eigenvalues as measures of the total variability explained by each principal component, showing a couple of **stiff** directions in the parameter space. For each group (survivor or non-survivor), ten thousands parameter sets were randomly sampled from the five million parameter sets of pool and analyzed based on the inverse correlation matrix (approximate Hessian). The coefficients of the linear combinations of the original variables that generate the first (**B**), second (**C**), and third (**D**) principal components were compared between the survivor and non-survivor populations. These comparative plots reveal the differences of the individual parameter's contributions to the first three largest principal components between two groups. The parameters that are contributing up to 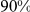 in each principal component were indexed.

The model parameters and their correlations reflect the influence of internal and external factors on the model. To explore potential factors that can differentiate the survivor population from the non-survivor population, we analyzed and compared the model parameter ensembles derived from the survivor and non-survivor cohorts. In the setting of parameter estimation of complex models, it has been well recognized that model behavior is very sensitive to a few directions called *stiff* directions and insensitive to the others, so-called *sloppy* directions [22]. The *stiff* directions represent parameter combinations that are well constrained by the experimental observations and affect the cost function the most. These *stiff* and *sloppy* directions are identified by performing principal component analysis (PCA) on the Hessian of the cost function. Hessians were approximated by inverting the empirical covariance matrices of the survivor and non-survivor parameter ensembles [17]. The eigenvalues measure the total variability explained by each principal component and the eigenvectors corresponding to the largest eigenvalues denote the stiffest directions in the shape of the cost manifold, or the parameter combinations that best characterize the inflammatory response. A few principal components carry most of the observed variability, and therefore the eigenvectors corresponding to these eigenvalues also display the most difference between the survivor and non-survivor ensembles (Figure 4 (**A**)). Contrasting eigenvectors between the survivor and non-survivor ensembles illustrates key differences between the survivor and non-survivor ensembles.

The coefficients of the linear combinations of the original variables contributing to the first three largest principal components, which represents the individual parameter's contributions to the corresponding principal components, are illustrated in Figure 4 (**panels B, C, D**). Large absolute magnitude of a coefficient (in either survivor, x-axis, or non-survivor, y-axis) imply major contributions of that parameter to the principal component direction. Deviations from the diagonal line indicate the parameters that lead to different behaviors between the survivor and non-survivor models. These comparative plots between the survivor and non-survivor populations reveal parameter subsets that characterize mechanisms associated with mortality in the non-survivor population. This is especially applicable to the first principal components, but not exclusively given the non-negligible nature of other components to total variability in Figure 4 **A**. In Figure 4 **B**, parameters 1 (PI activation threshold by bacteria), 3 (PI inhibition threshold by AI), and 22 (time constant of neutrophil sequestration) that are aligned with the diagonal line are major contributors to the first principal components of both populations. It is also noted that most components lies along this diagonal, suggesting that that the immune response to sepsis triggers similar mechanisms, in first approximation, in survivors and non-survivors. On the other hand, parameters 5 (AI activation threshold by PI) and 32 (time constant of damage resolution) are off-diagonal and contribute more to the non-survivor ensemble than the survivor ensemble, suggesting that unbalanced anti-inflammatory response and damage resolution contribute to death. In addition, parameters 19 (time constant of neutrophil migration to tissue) and 13 (time constant of blood neutrophil priming) are stronger contributors to the survivor ensemble, suggesting prompt neutrophil response to infection is a critical element for survival. Larger deviations were observed in the directions of principal components 2 and 3, as shown in Figure 4 **C** and **D**. Many of the parameter incongruences between survivor and non-survivor ensembles pertain to the regulation of pro- and anti-inflammatory regulators (1, 2, 3, 4, 5), indicating that differential dynamics is encapsulated in regulatory influences, rather than the main drivers of the inflammatory response.

#### Multivariate correlation analysis

**Figure 5:**
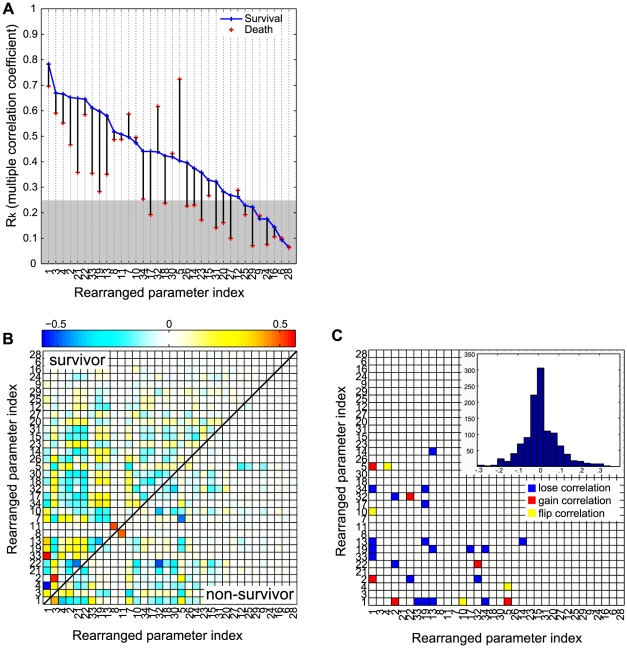
Multivariate correlation analysis of the parameter ensembles for survivor and non-survivor populations. For each group (survivor or non-survivor), ten thousands parameter sets were randomly sampled from the five million parameter sets of pool and analyzed based on the approximate Hessian matrix (the inverted empirical covariance). (**A**) The sample multiple correlation coefficient (R) as a measure of the strength of the single parameter's association with the other parameters. The shaded area represents the region below 0.25 that is generally interpreted as weak association. The partial correlations between two parameters are plotted for the survivor population (the upper triangular part in **B**) and the non-survivor one (the lower triangular part in **B**). The columns and the rows are rearranged according to the descending order of the multiple correlation coefficients in **A**. The parameter pairs whose correlations changed significantly between two groups are illustrated in **C**. The insert is the histogram showing the distribution of pairwise differences of the partial correlation coefficients in **B**.

While the correlation matrix provides merely bivariate information, the inverse correlation matrix reveals true multivariate interactions. The diagonal elements of the inverse of the correlation matrix are directly related to the multiple correlation of each parameter with the other parameters [23] (Equation 25 in the Materials and Methods). The coefficient of multiple correlation (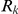) for the parameter 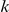, ranging from 0 to 1, quantifies the extent of multiple correlation between the parameter 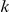 as the dependent variable and all of the other parameters as the independent variables: the larger the value of the multiple correlation coefficient, the stronger the association with all other parameters as a whole. Given that the parameter ensemble was estimated around the minima of the cost function, parameters with very small multiple correlation coefficients which are nearly independent of the other parameters behave like noises with respect to the cost function and could be set to constants in a model reduction exercise [24]. Figure 5 **A** compares the 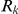 of the 34 model parameters for the survivor and non-survivor ensembles. The most notable feature of this comparative plot is that many parameters in the non-survivor population lose their multiple correlations with other parameters. This observation can be interpreted as potentially indicative of a loss of survival-associated regulation as characterized by weaker mechanistic cross-talk among inflammatory processes in the non-survivor ensemble. Interestingly, three parameters (5: AI activation by PI, 7: AI decay, 32: damage resolusion) showed increased multiple correlations, also suggesting these parameters represent mechanisms playing significant roles leading to death. These results are in line with the previous finding from PCA where prompt anti-inflammatory responses and damage resolution are critical element in survival.

Each element of the inverse correlation matrix is also directly related to a partial correlation [23]. Partial correlation analysis explores the linear relationship between two variables after adjusting for the effect of all other parameters, providing a more accurate reflection of the bivariate relationship between these parameters. The survivor ensemble included significantly more partial correlation than the non-survival ensemble (Figure 5 **B**). The parameter pairs that displayed statistically significant changes in their partial correlations between two ensembles (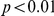) were categorized into three groups: *lose correlation*, *gain correlation*, and *flip correlation* (5 **C**). The parameter pairs in the *lose correlation* group significantly lose their partial correlations in the non-survivor population. *gain correlation* implies the opposite. The parameter pairs in the *flip correlation* group change their direction of correlations (positive or negative) between two population. Sixty percent of the significant changes are found in the *lose correlation* group, which confirms the previous finding that a loss of regulation (correlation) in the non-survivor population is associated with mortality. We also identified some key parameters that are frequently involved in the significant changes of pair correlations. Among them are parameters 19 (time constant for neutrophil migration), and 13 (time constant for neutrophil priming), suggesting again that coordinated neutrophil functions are critical for survival. The most significant loss of correlation was observed in the pair of parameters 13 and 1 (PI activation threshold by bacteria), suggesting that a positive correlation between the PI activation threshold and the time constant for blood neutrophil priming is essential for survival (Table 3). In other words, parameter sets which become more susceptible to systemic inflammation require more prompt neutrophil priming. The same line of interpretation can be inferred for the parameter pair 1 and 19.

**Table 3:** Parameter pairs whose partial correlations changed significantly between the survivor and non-survivor ensembles.

Parameter Index Pair		Partial Correlation Coefficient		
P1	P2	Survival	Death	Difference
**Lose Correlation**				
13	1	0.39081	0.076671	0.31414
33	1	0.57338	0.27114	0.30224
22	2	−0.37232	−0.086561	−0.28576
13	19	−0.39436	−0.11036	−0.284
19	1	0.33753	0.064503	0.27303
34	19	0.33952	0.070164	0.26936
14	13	0.2003	−0.051309	0.25161
19	17	0.28643	0.04326	0.24317
32	2	−0.24411	−0.021828	−0.22228
34	1	−0.25182	−0.036964	−0.21486
**Gain Correlation**				
32	22	−0.20802	−0.51495	0.30693
2	1	−0.026593	−0.2824	0.2558
5	1	0.073148	0.31583	−0.24268
**Flip Correlation**				
1	10	0.19069	−0.11887	0.30956
5	4	0.16015	−0.11315	0.2733

### Blood purification in sepsis: hypothetical mechanisms of action

**Figure 6:**
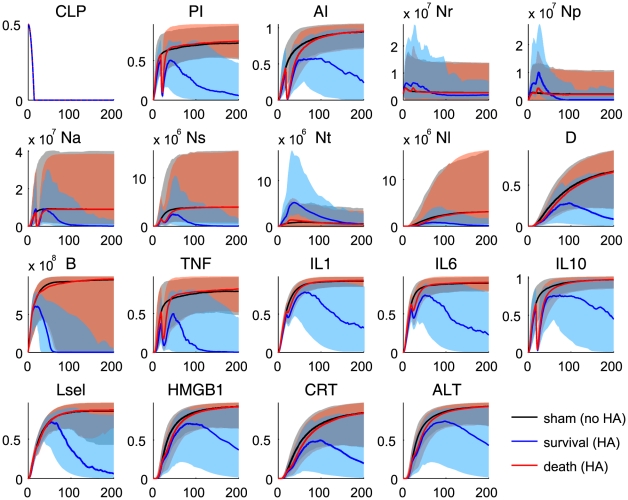
Hypothetical simulation of blood purification treatment. Ten thousands parameter samples from the non-survivor population were simulated with or without the extracorporeal blood purification device. Black denotes the sham case (without treatment), blue denotes the survivor case after the treatment, and red denotes the non-survivor case. The criterion of deciding survival was based on the last value of PI (threshold: 0.5). The shaded areas correspond to the 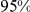 posterior limits of the model prediction uncertainty.

Although blood purification using HA results in important survival benefits in animal models of sepsis [11], [13], [25], our early attempts at understanding mechanisms through modeling suggested that cytokine removal alone was inadequate to explain the experimental findings [26], [27]. We confirmed this experimentally by scaling down cytokine removal below that which resulted in acute changes in circulating mediators and still found reduced organ injury and improved survival [12]. We then extended the model to include elimination of these inflammatory effector from the circulatory compartment by a hypothetical HA device. We hypothesized that the HA device adsorbs activated neutrophils (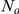) as well as pro- and anti-inflammatory mediators (PI and AI) from the circulation. To emulate reported experimental work [12], we simulated four hours of treatment starting at 18 hrs after CLP using a random set of 10000 parameter vectors sampled from the non-survivor ensemble. Output profiles were classified into survivor and non-survivor populations based on a 7-day value of 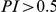. Figure 6 compares the non-survivor ensemble (shams) to survivors and non-survivor sub-ensembles obtained after treating shams with HA. About 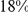 of the treated population (n = 1768) survived. We observe that enhanced neutrophil migration to the tissue (increased 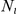) and reduced sequestered and lung neutrophils (decreased 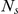 and 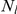) were all present in the survivor population after treatment, contributing to the improved outcome. These prediction results are consistent with our experimental observations [12], supporting that the proposed model could be used to guide future experiments and as a computational framework for generating hypotheses.

**Figure 7:**
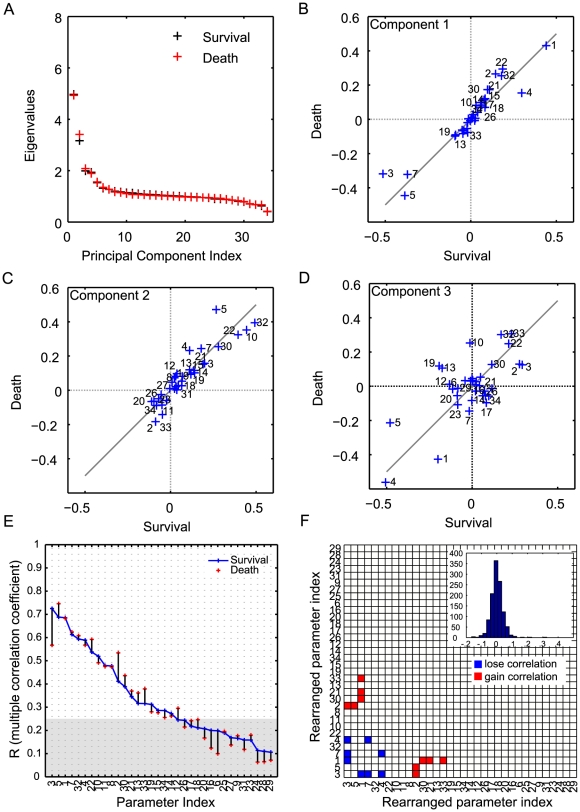
Multivariate correlation analysis of the parameter ensembles for survivor and non-survivor populations after blood purification (HA) treatment. (**A**) Eigenvalues plots for the two populations. Ten thousands parameter samples from the non-survivor population before HA treatment (sham) were classified into the survivor and non-survivor ones after the HA treatment. The coefficients of the linear combinations of the original variables that generate the first (**B**), second (**C**), and third (**D**) principal components were compared between the survivor and non-survivor populations after the HA treatment. (**E**) Multiple correlation coefficients of the two populations. (**F**) The parameter pairs with significantly changed correlations between two groups are illustrated. The notations are same as Figure 5 **D**.

To investigate which factors play an important role in successful HA treatment in a population that would otherwise die, we analyzed the two sub-ensembles of survivor and non-survivor after HA treatment in the same manner as the previous section (see Figure 7). We observed that systemic pro-inflammation related parameters are involved in most of the parameter pairs whose partial correlations changed significantly between the two sub-ensembles (Table 4). In particular, partial correlations involving parameter 3 (PI inhibition by AI) disappeared in the HA non-survivor population, suggesting a robust anti-inflammatory response is a critical factor for treatment success. Interestingly, when HA treatment was applied to a sample of the survivor ensemble, 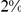 (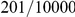) died according to our criteria, suggesting that there also exists a sub-population of survivors for whom treatment is actually harmful. Further analysis of univariate differences in parameter distribution between survivor and the harmed sub-population identified that non-survivors had larger PI inhibition by AI (parameter 3, 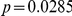) and faster PI decay rates (parameter 4, PI decay, 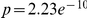).

**Table 4:** Parameter pairs whose partial correlations changed significantly between the survivor and non-survivor ensembles after HA treatment.

Parameter Index Pair		Partial Correlation Coefficient		
P1	P2	HA Survival	HA Death	Difference
**Lose Correlation**				
4	3	0.45487	0.129	0.32588
4	7	0.28884	0.062232	0.2266
7	3	−0.48204	−0.36471	−0.11733
3	1	0.49145	0.3787	0.11275
**Gain Correlation**				
2	3	0.1966	0.36179	−0.16519
5	2	0.22854	0.3917	−0.16316
33	1	0.1906	0.30413	−0.11353
1	21	−0.10914	−0.21761	0.10848
1	30	−0.079448	−0.18667	0.10723
**Flip Correlation**				
4	5	0.046393	−0.1257	0.17209

## Discussion

We have developed a coarse-grained phenomenological model of the inflammatory response to CLP-induced sepsis in rats that centers on dynamic interactions of distinct neutrophil phenotypes and fundamental effectors. The model simulations and reproduction of experimental data support our main hypothesis that systemic inflammation leads to heterogeneous circulating neutrophil subsets which contribute to differential fates of septic animals. The emergent properties observed in the *in silico* non-survivor population that systemically activated neutrophils lose their chemotactic ability to the infectious focus and instead become trapped in narrow lung capillaries comply with biological domain knowledge [8], [28].

Given that the experimental sepsis model was performed with a high consistency in a standardized manner to minimize extrinsic noises [29], the heterogeneity of sepsis severity can be assumed to originate from intrinsic (genetic and epigenetic) differences in rats. In order to investigate the underlying differences, we identified ensemble models for two distinct severity populations, *survivor* and *non-survivor*. The network structure and initial conditions of the model were assume to be identical in all populations. Therefore, the heterogeneity in populations is represented by differences in parameter distributions within ensemble. This population-based ensemble approach allows us not only to assess parametric uncertainty, but also to characterize differences in parameters between distinct populations. Multivariate analyses of the population ensembles suggest that balanced regulation of the pro-/anti-inflammations and coordinated neutrophil functions play important roles in survival. The non-survivors are characterized by a loss of dynamic features in survival-associated regulation of inflammatory responses. First, delayed anti-inflammatory response and damage resolution contribute to mortality. Second, prompt neutrophil priming and migration to the infectious focus before uncontrollable systemic inflammation develops are critical for survival. These *in silico* findings underline the importance of timely diagnosis and treatment of sepsis in clinical practice.

With the extremely complex nature of sepsis in mind, determining the precise inflammatory status can be very useful to start timely and specific treatment. Patients with hyper-responsive inflammatory states will benefit from limiting their inflammation, whereas others with hypo-responsive states would be better treated by boosting inflammation [1]. However, assessing the precise inflammatory status still poses a significant challenge. Plasma levels of cytokines may not be sufficient to define accurately the inflammatory state because pro- and anti-inflammatory mediators increase simultaneously in septic patients and animals [30]. In addition, the dynamic changes of cytokine levels in severely septic patients are not clearly consistent with the course of sepsis [31].

Our simulation work therefore supports the notion that an evaluation of cellular function would be a better method than measuring soluble mediators alone to define the precise inflammatory response and should be targeted clinically [32]. Among the effector cells in the septic response, neutrophils are critical elements of the innate immune response to the infection. Recent studies have reported several molecular mechanisms for dysregulated neutrophil trafficking over the whole spectrum of neutrophil migration. The systemic activation of TLR4 results in down-regulation of neutrophil rolling on endothelial cell surface and migration to the tissue [6], [33]. Patients with a deficiency of leukocyte adhesion molecules show easier bacterial infection and sepsis development [34]. Gaseous molecules such as nitric oxide (NO) and peroxynitrite (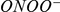) downregulate neutrophil migration by reducing leukocyte adhesion and migration [35], [36]. Cunha *et al* showed that excessive production of NO during sepsis induced by Toll-like activation reduces the expression of the chemokine receptor CXCR2 in circulating neutrophils and contributes to the impairment of neutrophil migration [37]. These results provide experimental evidence that altered neutrophil phenotypes in the circulation contribute to the pathogenesis of sepsis and its mortality. More precisely then, our study suggests that a phenotypic characterization of circulatory neutrophils would be an effective way to determine the inflammatory status and guide future therapeutic strategies. Changes in the relative proportion of neutrophil phenotypes and their absolute numbers, as measured in the circulation, could constitute an effective early marker of disease progression or the therapeutic effect of an intervention on infection control and downstream organ dysfunction. In view of the multiple factors modulating neutrophil functions, using multiple markers to quantify the differential expression of neutrophil receptors looks promising [8], [9], [38].

Considering the highly heterogeneous population of patients with sepsis, identifying a sub-population of patients that is most likely to benefit from a specific intervention is potentially of great benefit in the design of interventional trials or bedside therapeutic decisions. Mathematical modeling can be a useful framework toward this goal. Our model simulations coupled to a hypothetic HA device model generated a subset of animals that survived after HA treatment and their survival features were characterized by their parametric description. In order to make this *in silico* work meaningful for translation, the parametric descriptions should be translated into measurable biological or physiological phenotypes, that is predictive biomarkers. The key idea is that population dependent parameter distributions reflect the heterogeneity of the treatment efficacy. A distribution of physiologically interpretable model parameters and states inferred from the patient information may be able to serve as early-stage markers for identifying a sub-population that can be benefited from a certain treatment option. Further experimental investigations are warranted to validate our computational findings. Furthermore, the experimental and clinical relevance of our analysis on HA treatment simulations are limited by a simplistic HA device model which was not calibrated by experimental data. We recently developed a more realistic HA device model calibrated by *in vitro* experimental data [39]. Coupling to a calibrated HA device model and rigorous analysis of the effects of HA treatment are currently underway.

In conclusion, the ensemble models constructed herein in order to explore heterogeneity in distinct sepsis severity populations provided useful insights as to key pathologic mechanisms associated with mortality in sepsis. The population-based ensemble approach can be extended to explore critical mechanistic differences between different pathologies within a same context of disease. One could therefore apply the method to investigate differences in chronic/acute, old/young, races, or any other “natural” groups. The population-based computational framework holds promise as a tool for integrating domain knowledge and experimental data into a quantitative assessment of population dynamics.

## Materials and Methods

### Experimental protocol

The CLP-induced sepsis experimental protocol is a recommended proxy for human sepsis, where the infection spreads beyond a local focus, resulting in systemic symptoms, septic shock and a high mortality [29], [40]. The experiments were designed to evaluate long-term (one week) survival in a model of sepsis that resulted in a mortality rate similar to that observed clinically. Following approval by the Animal Care and Use Committee of the University of Pittsburgh, the CLP procedure was modified (25% ligated length of cecum and 20-gauge needle, two-puncture) in rats to induce less lethal sepsis compared to that which we have described previously [25]. Plasma cytokines (tumor necrosis factor (TNF), interleukin(IL)-1*_β_*, IL-6 and IL-10), high mobility group box1 (HMGB1), creatinine (CRT) and alanine aminotransferase (ALT) were measured from 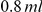 blood samples from 23 rats at 18, 22, 48, 72, 120, 144, and 168 h after CLP. Each cytokine measurement data was natural log transformed and normalized by its maximum value across all time points for all animals. Other measurements were also normalized by their maximum values. Seven rats out of the total population survived up to 7 days, being considered as the survivor population; the remaining 16 animals comprised the non-survivor population.

### Model formulation and simulation

The network components and interactions of the model were compiled from available information in the literature and the general domain knowledge about the acute inflammatory response. Ordinary differential equations (ODEs) governing the phenomenological signaling interactions in the network were formulated based on the standardized steps presented in [41], [42]. We wished to represent the qualitative interactions in the system as a Boolean model and then transform the logic operations into a system of ODEs. We chose HillCube ODEs as continuous homologues of the Boolean interactions, in which model parameters were regularized as one of three types: Hill coefficient(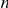), half maximal activation constant(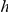), or time constant (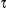). Exceptions were made for model parameters that should be constrained by mass action kinetics.

#### Bacteria

The following equation describes the population of bacteria (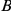) in the peritoneum, the local site of infection.
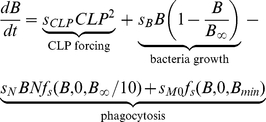
(1)where the function 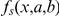 is linear function of 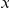 between its saturation points 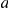 and 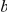. CLP induces bacteria in the peritoneum assuming peritoneal bacteria increase in number at the constant rate of 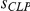. The unit interval continuous variable 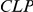 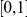 stands for the severity grade of the CLP operation that is known to depend on the puncture size and the length of ligation [43]. The logistic growth term assumes that bacteria increase in number at the growth rate 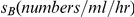 until bacterial count approaches the carrying capacity (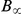) at which point growth slows. Tissue neutrophils migrate from the blood circulation and kill Bacteria at the rate 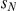 upon encountering. The function 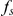 was introduced to describe the saturation of neutrophil-killing capacity at a high bacterial concentration [44]. The last term was introduced in order to account for the bacterial removal by peritoneum-resident macrophages even before effective neutrophil migration into the infection site. 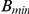 was set to 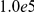 (CFU/ml), assuming resident macrophages can remove up to this amount of bacteria without migrated neutrophil before bacteria increase in number significantly.

##### Neutrophils

The following set of equations describe multiple populations of neutrophils; resting blood neutrophils 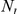, primed blood neutrophils 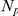, activated blood neutrophils 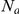, neutrophils that migrated to the peritoneum 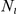, neutrophils sequestered in the lung capillaries 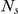, and migrated lung neutrophils 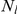.
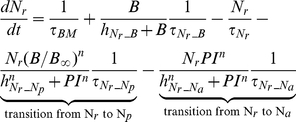
(2)

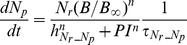
(3)

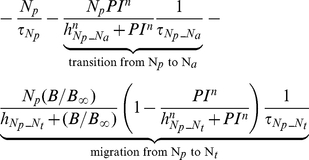
(4)

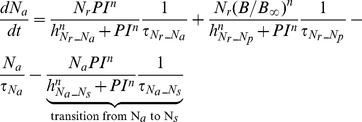
(5)

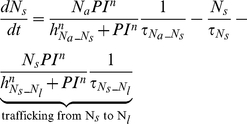
(6)

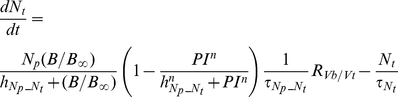
(7)

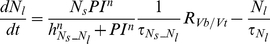
(8)


The bone marrow reserve of neutrophils releases mature neutrophils into the circulation at the rate of 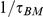 under homeostatic conditions and circulating neutrophils die at the rate of 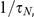 by undergoing normal apoptosis. During inflammatory reactions, neutrophils are rapidly mobilized from the bone marrow, and this rate is nonlinearly dependent on the bacteria burden. The variable 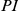 stands for the extent of the systemic inflammation that are coarse-grained to conceptually expresses systemically acting pro-inflammatory factors in circulation. It is hypothesized that neutrophils in the circulating pool exist in three states: resting, primed, and activated. Resting neutrophils (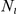) are first *primed* (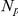) by interacting with inflammatory mediators before migrating into tissue (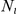). When neutrophils become activated (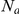) in the blood compartment, they lose their chemotactic ability into the infected tissue by downregulating their chemotactic receptors and become sequestered in the lung capillaries (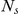). The mass-action based transitions of neutrophils from a resting state to a primed state and/or an activated state were controlled by Hill functions that are nonlinearly dependent on the local infection level (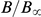) or the systemic inflammatory status 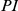. The term, 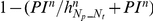 in the equation (4) and (7) was introduced to represent a collective response of several processes that have been reported to contribute to the impairment of neutrophil migration during severe sepsis [6], [15]. All Hill coefficients are identically set to three for parametric simplicity.

##### Inflammation and damage

The following set of equations describe three conceptual unit interval variables in the network: 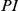, 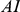, and 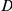. As a counteracting variable of 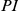, 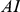 describes the level of the anti-inflammation corresponding to systemically acting anti-inflammatory mediators such as IL-10. 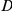 is a course grained representation of integrated tissue damage expressing neutrophil-induced tissue injuries that may lead to organ failure.
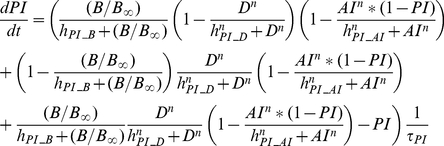
(9)

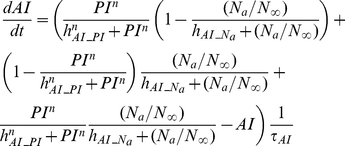
(10)

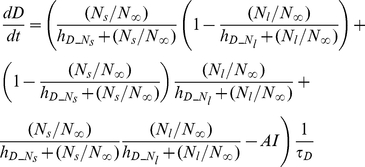
(11)

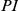
 can be activated by bacteria 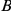 or damage factor 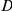, but it is inhibited by 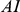. However, plasma cytokine measurements in severe sepsis have shown that a high level of anti-inflammatory activity coexists with the increased activation of the inflammatory response, reflecting the fact that anti-inflammatory effect cannot fully counteract the pro-inflammatory stimuli beyond a certain high level of systemic inflammation [30]. Therefore a partial inhibition of 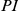 by 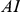, depending on the level of the systemic inflammation, was assumed by introducing the term 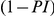 which moderates the effect of 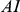 in high 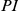.

##### Observations

Explicit interactions among observational state variables were not constructed due a lack of kinetic and causal information available. Instead, we hypothesized that pro(anti)-inflammatory blood cytokines (TNF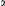, IL-1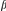, IL-6 or IL-10) levels are nonlinear reflections of a higher-level pro(anti)-inflammatory status with sigmoidal relationships.
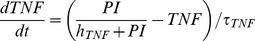
(12)

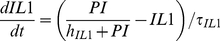
(13)

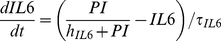
(14)

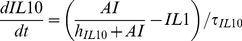
(15)


Damage related markers (HMGB1, CRT, ALT) are also nonlinearly mapped from a higher-level state variable, which represents the systemic damage abstractly.
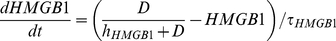
(16)

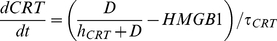
(17)

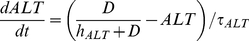
(18)L-selectin, known to be a marker of neutrophil activation, was quantified by mapping from the rate of neutrophil activation (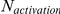) which is quantified by the sum of the transitions from 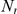 to 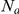, 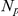 to 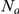, and 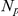 to 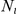.
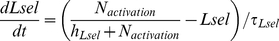
(19)


##### Hypothetic HA mechanisms of action

The hypothetic mechanisms of action of the blood purification was implemented by assuming the HA device eliminates only three components in the circulation: activated neutrophils (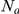), pro-inflammatory mediators (PI), and anti-inflammatory mediators (AI) during the treatment period (from 18 hours to 22 hours after CLP). The rate of elimination was modeled by a Hill equation (Hill coefficient 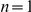) that are nonlinearly dependent on the concentration of each component.
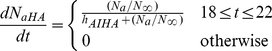
(20)

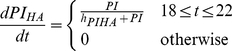
(21)

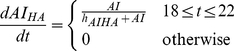
(22)


In the setting of HA simulations, these terms were subtracted from equations 5, 9 and 10 respectively.

### Implementation of Metropolis Monte Carlo sampling

The five parameter sets randomly sampled from the baseline parameter distribution in Table 1 were used as starting points to generate Markov Chain Monte Carlo samples based on the Metropolis Algorithm [45], [46]. A uniform (non-informative) prior distribution over the range specified by the lower and upper bounds of parameters was chosen. The prior ranges were made very wide to include all plausible values. For example, the prior ranges for threshold parameters were set to 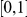 and the ones for time constants were set to 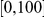. The goal is then to draw samples in the accessible parameter space from the posterior target distribution 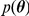, which was taken to be proportional to the likelihood 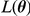, the probability that our model with parameters 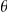 would generate the observed data 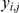. The difference between the measured and simulated value of species 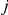 at time 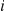 was quantified by the cost function, or the weighted sum of squared residuals,
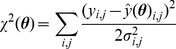
(23)with 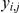 and 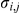 denoting the mean and standard deviation of the measured value of species 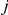 at time 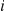 and 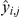 denoting its simulated value. Assuming that the measurement noises represent Gaussian random measurement errors, the target distribution is as follows.
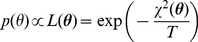
(24)which is an analogy to a Bolzmann distribution with energy 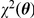 and temperature 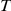. The temperature 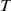 was used to tune the rate of acceptance of candidate parameter sets as the Markov chain is constructed, where the acceptance ratio increases with increasing temperatures. The proposal density that generates a new candidate set of parameters using current values (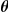) was chosen to be a normal distribution centered at the current point, 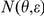. The parameter 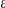 was also used to tune the acceptance ratio of candidate samples with smaller values increasing the ratio. We tuned 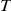 and 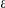 to get a reasonable convergence with the acceptance ratio being around 0.25 [47].

The numerical integration of the systems of ODEs described above was implemented by using the SUNDIALS package (https://computation.llnl.gov/casc/sundials/main.html). All other algorithms used for this work were implemented in the MATLAB version 7.10.0.499 (The MathWorks). The source code used in generating results is available at http://code.google.com/p/source-code-sepsis-model/.

### Multivariate analysis of the parameter ensemble

The *stiff* directions in the parameter space can be identified by the principal component analysis of the Hessian matrix [17]. Instead of using the computationally expensive Hessian matrix, we used 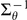 the inverse of the correlation matrix of the parameter ensemble, with the understanding that in a maximum likelihood estimation setting the covariance matrix of the parameters can be approximated by the Hessian matrix of the likelihood function [48]. An eigenvalue decomposition of 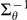 allowed us to obtain the information about stiff (large eigenvalue) directions in the parameter space.

The inverse of the correlation matrix can also be used to extract valuable information in multivariate data [23]. The basic formulas for computing the multiple correlation coefficients and the partial correlation coefficients are as follows. The diagonal elements of 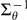, 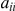, are related to the multiple correlation between the parameter 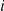 and all other parameters.
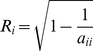
(25)The partial correlation between the parameter 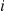 and the parameter 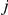 controlling all other variables can be calculated as
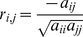
(26)

