# Correction: Ensemble Models of Neutrophil Trafficking in Severe Sepsis

**DOI:** 10.1371/annotation/4ca315e7-1219-46fd-af5c-c29b8e6ecf93

**Published:** 2012-08-14

**Authors:** Sang Ok Song, Justin Hogg, Zhi-Yong Peng, Robert Parker, John A. Kellum, Gilles Clermont

The first author's name was spelled incorrectly. The correct name is: Sang Ok Song

